# In Vivo Effects Of Traditional Ayurvedic Formulations in *Drosophila melanogaster* Model Relate with Therapeutic Applications

**DOI:** 10.1371/journal.pone.0037113

**Published:** 2012-05-14

**Authors:** Vibha Dwivedi, E. M. Anandan, Rajesh S. Mony, T. S. Muraleedharan, M. S. Valiathan, Mousumi Mutsuddi, Subhash C. Lakhotia

**Affiliations:** 1 Cytogenetics Laboratory, Department of Zoology, Banaras Hindu University, Varanasi, India; 2 Arya Vaidya Sala, Kottakkal, India; 3 Manipal University, Manipal, India; 4 Department of Molecular and Human Genetics, Banaras Hindu University, Varanasi, India; University of Massachusetts Medical School, United States of America

## Abstract

**Background:**

Ayurveda represents the traditional medicine system of India. Since mechanistic details of therapy in terms of current biology are not available in Ayurvedic literature, modern scientific studies are necessary to understand its major concepts and procedures. It is necessary to examine effects of the whole Ayurvedic formulations rather than their “active” components as is done in most current studies.

**Methods:**

We tested two different categories of formulations, a *Rasayana* (*Amalaki Rasayana* or AR, an herbal derivative) and a *Bhasma* (*Rasa-Sindoor* or RS, an organo-metallic derivative of mercury), for effects on longevity, development, fecundity, stress-tolerance, and heterogeneous nuclear ribonucleoprotein (hnRNP) levels of *Drosophila melanogaster* using at least 200 larvae or flies for each assay.

**Results:**

A 0.5% (weight/volume) supplement of AR or RS affected life-history and other physiological traits in distinct ways. While the size of salivary glands, hnRNP levels in larval tissues, and thermotolerance of larvae/adult flies improved significantly following feeding either of the two formulations, the median life span and starvation resistance improved only with AR. Feeding on AR or RS supplemented food improved fecundity differently. Feeding of larvae and adults with AR increased the fecundity while the same with RS had opposite effect. On the contrary, feeding larvae on normal food and adults on AR supplement had no effect on fecundity but a comparable regime of feeding on RS-supplemented food improved fecundity. RS feeding did not cause heavy metal toxicity.

**Conclusions:**

The present study with two Ayurvedic formulations reveals formulation-specific effects on several parameters of the fly's life, which seem to generally agree with their recommended human usages in Ayurvedic practices. Thus, *Drosophila*, with its very rich genetic tools and well-worked-out developmental pathways promises to be a very good model for examining the cellular and molecular bases of the effects of different Ayurvedic formulations.

## Introduction

Ayurveda represents the traditional medicine system of India which is widely practiced uninterruptedly at least from beginning of the Buddhist period in India. It continues to be a vibrant system of health care for millions, with over twenty thousand physicians being trained every year and which supports an industry producing drugs worth Rs. 6000 crores (US $1.3 billion) a year. By definition, Ayurveda signifies “knowledge relating to life”, and regards diseases and medicine as no more than facets of the variegated theme of life. Ayurvedic texts like *Sushruta Samhita* [1] divide the discipline into eight branches, of which the rejuvenating *Rasayana* therapy aims at promotion of long life, enhancement of physical and mental strength, and strengthening of resistance against the infirmities and ailments of old age. *Rasayana* therapy calls for ethical living in conjunction with intramural or extramural protocols involving life style, diet, cleansing procedures and the intake of medicinal formulations. The intramural as well as extramural methods of *Rasayana* therapy require oral administration of drugs, which are mostly based on plant products but may also include drugs derived from animal and mineral/metal sources. Improvement in nutritional status and better qualities of body tissues (*dhatus*) are believed to lead to a series of secondary attributes like longevity, immunity against disease, improved mental and intellectual competence etc [2]. Etymologically, *Rasayana* implies supply of the nutrient sap (*Rasa*) resulting from the digestion of food to the target (*ayana*) body tissues. As described in *Charak Samhita* [3], *Rasayanas* are believed to augment the transport and supply of “*Rasa*” to the tissues.

The two major groups of Ayurvedic drugs are *Kasthoushadhies* (herbal preparations) and *Rasaoushadhies* (Herbo-bio-mineral-metallic preparations). The *Bhasma*, belonging to the *Rasaoushadhies* group, has a metallic base but ordinarily does not contain active metal. The metal is converted into an ash or oxide and is usually in the form of an organo-metallic compound formed with a number of organic materials used for trituration as *Bhavana Dravya* [2]. Many a times, Ayurvedic drugs are administered orally with ‘*Anupana*’, a vehicle material like honey, sugar, jaggery, Ghee, milk, warm water, juice of some medicinal herbs etc. *Anupana* as a medium of administration improves acceptability and palatability and helps in absorption of the main drug; additionally, it may also act as early antidote.

The ancient Ayurvedic literature available today does not elaborate the effect/s of any therapy in terms of our current understanding of biology/physiology. Given the antiquity, extensive use in health care, and the globally growing popularity of Ayurveda, it would be interesting and appropriate to initiate a new class of studies which apply the rigorous methods of modern science to understand its major concepts, procedures and mechanistic aspects of the products [4]. Although, there has been an increased interest in traditional and herbal medicine systems in recent times, most studies have used specific extracts or “active principles” derived from herbal or other traditional drugs/formulations. Since the Ayurvedic medicines/formulations are complex integrated derivatives involving several specific preparatory steps, studies using isolated active compounds may not really provide full insight into the efficacy or mode of action of the traditional formulations. In order to undertake scientific investigations on action/s of Ayurvedic drugs/formulations using experimental animals, there is an urgent need to develop good model systems which can permit in depth studies on the *in vivo* effects and mechanisms of actions of different Ayurvedic formulations.

With a view to get insight into the cell biological/biochemical/genetic bases of actions of the Ayurvedic formulations, we are using the fruit fly, *Drosophila melanogaster*, as a model. The advantages of the fly model as an experimental model, especially for examining the diverse factors that affect life-history traits and for understanding complex human disorders, are well known [5], [6], [7], [8], [9], [10]. As a first approach in this direction, we used *Amalaki Rasayana*, a herbal derivative, and *Rasa-Sindoor*, an organo-metallic derivative of mercury, to examine if these formulations indeed affect some of the basic biological life parameters in the fly model. *Amalaki Rasayana* (AR) is a prominent drug in Ayurvedic classics like *Charak Samhita* [3] and *Ashtang Hridaya* [11] and continues to be widely used in view of the claim that it enhances life expectancy, body strength, intellect, fertility and gives freedom from illness. *Rasa-Sindoor* (RS), on the other hand is indicated, singly or in combination with other *Anupana*/formulation in a wide variety of disorders including chronic and recurrent infections (pneumonia/bronchitis), fistula-*in ano*, rheumatological diseases especially those of auto-immune origin, sexual and general debility and benign and malignant neoplasms [12], [13].

AR is a *Rasayana* prepared from fruits of Amla or Indian gooseberry (*Phyllanthus emblica*, synonym *Emblica officinalis*). RS, on the other hand, is an organo-metallic *bhasma* in the form of mercuric sulphide with some other elements also present in micro/trace quantities [14]. RS has a crystalline nature with crystal size ranging from 25 to 50 nm, close to the nano-crystalline materials [14], [15].

In the present report we have standardized the amounts of these two formulations for supplementation in the standard fly-food that have demonstrable effects on several general physiological parameters like life span, development time, fecundity, thermo-tolerance, starvation resistance etc. In our study, we did not attempt to characterize the “active principles” in the AR or RS since in Ayurveda, these formulations are believed to act as a whole, rather than through one or more of their compound/s in isolation. Our results show that feeding of the fly larvae and/or adults on food supplemented with non-toxic levels of the two very different classes of Ayurvedic formulations, viz., the herbal-derived AR and the organo-metallic RS, have distinct supplement-specific effects, which in general appear to be in agreement with those described in Ayurvedic literature. Thus, *Drosophila*, with its very rich genetic tools and well worked out developmental pathways promises to be a good model for examining the cellular basis of the effects of different Ayurvedic formulations.

## Results

### Standardization of dosages of AR and RS which affect life history parameters of *Drosophila*

**Figure 1:**
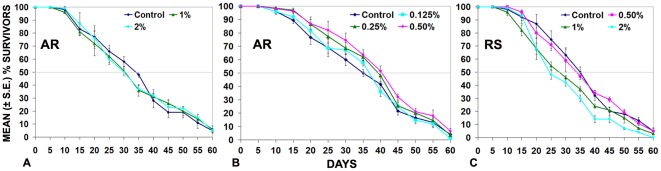
Viability assay of flies reared since embryo hatching on various concentrations of AR or RS supplemented or regular food. The survival curves of flies reared since the 1^st^ instar stage on food supplemented with 1% (green) or 2% AR (light blue) are shown in **A**, for those grown on food supplemented with 0.125% (light blue) or 0.25% (green) or 0. 5% AR (red) are shown in **B; C** shows survival curves for those grown on food supplemented with 0.5% red) or 1% green) or 2% RS (light blue). Each of these also shows the survival curve for flies reared in parallel on regular food (Control, dark blue). Survival curves are based on observations on 200 (8 replicates of 25 flies each) adult flies in each set. Although surviving flies were counted on daily basis, the data are presented for 5 day intervals for the sake of convenience. The vertical bar at each data point indicates the ±S.E. of mean % survivors in the 8 replicates. Median life span is estimated as the day till which 50% (horizontal gray line at 50% on Y-axis) of original flies were still surviving.

In initial experiments, we fed wild type larvae on food supplemented with 1 or 2% of the AR (complete with honey and Ghee) to see effects on development and life span. While the development was not significantly delayed, the median life span of flies fed on 1 or 2% AR supplemented food since the 1^st^ instar stage was reduced compared to those reared on regular food (Fig. 1A, Table 1). In view of the apparently toxic effects of higher concentrations, we used 0.5%, 0.25% or 0.125% AR supplemented food for the median life span assay. Flies fed, since 1^st^ instar larval stage, on food supplemented with lower concentrations of AR showed a dose-dependent increase in the median life span, with 0.5% AR supplemented food resulting in maximal increase (40.4 days compared to 36 days for flies reared on regular food; Fig. 1B, Table 1). Supplementing fly food with only honey (0.286%) or only Ghee (0.072%) or with honey plus Ghee (0.36% in 1∶0.25 ratio) at dosages equivalent to that in 0.5% AR supplemented food, did not significantly affect the median life span (data not presented). Therefore, in all subsequent experiments, we used 0.5% AR supplemented food.

**Table 1:** Median Life Span of *Oregon R^+^* flies fed on regular or AR or RS supplemented food.

Food supplement	Median life span of flies under different feeding regimes (in days)					
	Control	0.125%	0.25%	0.5%	1%	2%
**Amalaki Rasayana**	36.0	37.4**	38.8*	40.4*	30.4*	30.0*
**Rasa-Sindoor**	35.1	35.2**	35.0**	35.3**	30.1*	25.4*

N = 200 for each feeding condition.

* P<0.001 when compared with the corresponding control (reared on regular food) set of flies;

** P>0.05 when compared with the corresponding control.

In the case of RS, initially 1^st^ instar larvae were fed on regular food supplemented with 2%, 1% or 0.5% RS to see effect on life span of adult flies. While 1% or 2% RS supplement reduced the median life span, 0.5% had no significant effect (Fig. 1C, Table 1). Lower concentrations of RS also did not affect the median life span (data not shown). Therefore, 0.5% RS was used in all subsequent experiments.

### Rearing of larvae on 0.5% AR or RS supplemented food marginally hastens pupation and fly eclosion by a few hours

**Figure 2:**
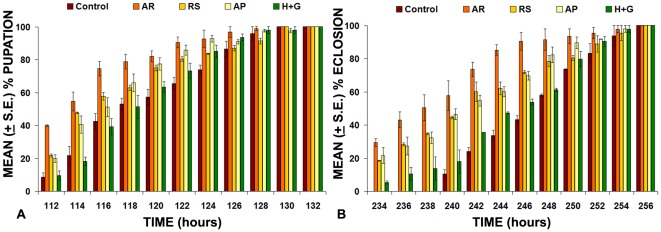
Feeding of *Oregon R^+^* first instar larvae on food supplemented with 0.5% of AR or RS hastens pupation (A) and fly eclosion (B). The bars represent mean (± S.E.) of 8 replicates (N = 25 in each replicate; total N for each feeding regimen = 200). Numbers of pupae (**A**) or flies (**B**) in a given sample were monitored every two hours between 112–132 and 234–256 hr (X-axis), respectively, and expressed as % of larvae that had pupated or flies that had eclosed by the given time period. Control – regular food, AR – 0.5% AR supplemented food, AP- 0.143% Amalaki Powder supplemented food. RS – 0.5% RS supplemented food and H+G – Honey plus Ghee (same proportion as in 0.5% AR) supplemented food.

The 1^st^ instar larvae that had hatched within one hour interval from the same batch of wild type eggs were distributed to plates with i) regular food (Control), ii) 0.5% AR supplemented food, iii) food supplemented with honey or Ghee or honey+Ghee or the triturated Amalaki powder only in the same proportion as in 0.5% AR supplemented food or iv) 0.5% RS supplemented food. The supplements in iii) served as additional controls for the AR-supplemented food (ii). The developmental assay revealed that larvae reared on 0.5% AR or 0.5% RS supplemented food developed a little faster since pupation in these dishes started a few hours earlier than in those having regular food or honey plus ghee supplemented food (Fig. 2A; Table 2). It is significant that the time of earliest and last pupating larvae is consistently shifted a few hours earlier than in those growing in parallel on the regular food (see Table 2). Adult flies also emerged earlier in AR or RS fed samples. The faster development was more apparent in the AR fed larvae (Fig. 2B). Supplementing the food with honey and Ghee (H+G in Fig. 2, Table 2) or only Ghee or only honey (not shown) did not affect the normal rate of development. However, supplementing food with the triturated Amalaki powder only (0.143%, AP in Fig. 2) also hastened the development but it was less than that seen with the whole AR supplement (Table 2).

**Table 2:** Median pupation and adult eclosion times following rearing on regular or AR or RS supplemented food.

Food supplement	Median Pupation time (in hours) ±S.E.	Median Eclosion time (in hours) ±S.E.
**Regular (Control)**	116.1±2.2	246.1±1.6
**Honey + Ghee**	116.1±2.7**	245.6±1.5**
**Amalaki Powder**	114.3±1.9*	240.6±1.7*
**Amalaki Rasayana**	112.1±2.3*	236.7±3.3*
**Rasa-Sindoor**	114.1±2.1*	240.2±2.9*

N = 200 for each feeding condition (8 replicates of 25 each).

* P<0.001 when compared with the corresponding control (reared on regular food) set of flies;

** P>0.05 when compared with the corresponding control.

### Dimensions of late 3^rd^ instar larval salivary glands and their polytene nuclei are increased following feeding on formulations

**Figure 3:**
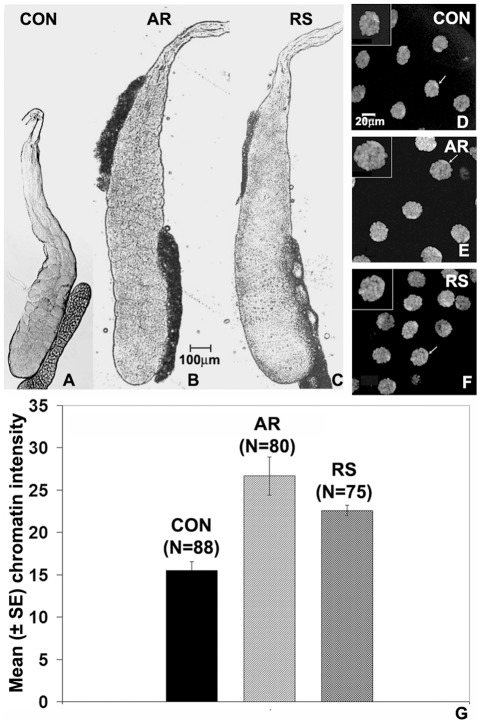
Larval salivary glands attain greater size following feeding on 0.5% AR (B) or RS (C) than in normally fed larvae (A) of same age (spiracle eversion stage). Individual polytene nuclei are also bigger in larvae reared on AR- (**E**) or RS-supplemented (**F**) food than in those fed on regular food (**D**); nuclei marked with arrows in **D**–**F** are shown at higher magnification in insets (also see Table 2). Histograms in **G** show the mean (±S.E.) DAPI fluorescence (in arbitrary fluorescence units on Y-axis) in distal polytene nuclei in salivary glands from larvae reared on regular (CON) or formulation (AR or RS) supplemented food (numbers of nuclei examined in each case are indicated in parentheses above the respective bars. Scale bar in **B** applies to **A**–**C** while that in **D**, applies to **D**–**F**.

In order to see if the AR or RS-supplement in food affected internal larval organs, we examined the different internal organs of late 3^rd^ instar larvae. Various organs like the gut, imaginal discs, brain ganglia etc in larvae reared on the formulation supplemented food appeared generally comparable in size and disposition to those in larvae fed on regular food. Their SG, however, appeared somewhat larger in the formulation fed larvae. Therefore, we compared the dimensions (width and length) of SG of late third instar (spiracle eversion stage) larvae that were fed on regular (Fig. 3A) or 0.5% AR (Fig. 3B) or 0.5% RS (Fig. 3C) supplemented food since hatching. It is interesting that the length and width of the SG are significantly enhanced (Table 3 and Fig. 3A–C) in larvae grown on formulation supplemented food. The number of cells in each of the larval salivary glands is fixed during embryonic stage and the subsequent growth of this tissue occurs through endo-replication cycles that are highly regulated in relation to the anatomical location of each nucleus in the gland [16], [17], [18]. Therefore, we wanted to know if the increased dimensions of these glands correlated with change in nuclear size and increased DNA content. In order to obtain SG of same age, larvae that had just everted their anterior spiracles were selected and immediately dissected in Poels' salt solution (PSS) [19]. Measurement of diameters of nuclei in the posterior most 5–7 cells in these glands revealed that the nuclear size is significantly greater in the AR- (Fig. 3E) or RS-fed (Fig. 3F) larval SG compared to that in normally fed control larvae (Fig. 3D). Measurement of DNA-specific DAPI fluorescence of distal nuclei in salivary glands from larvae that had just begun the spiracle eversion (Fig. 3D–F, Table 3) revealed that increased nuclear size is paralleled by increase in the DNA content in these polytene nuclei. These effects are more pronounced in AR-fed larvae than in those receiving the RS supplement (Table 3 and Fig. 2G). These observations show that while the larvae reared on formulation supplemented food take a few hours less to pupate than those reared on regular food (see Fig. 2 above), the polytene nuclei in their SG undergo greater numbers of endoreplication cycles so that in parallel with increased DNA content per nucleus, the nuclear and SG dimensions also increase.

**Table 3:** Feeding on 0.5% AR or RS supplemented food during larval period increases dimensions of salivary glands of late 3^rd^ instar larvae.

Mean (±SE) dimensions (in µm)	Control	AR 0.5%	RS 0.5%
**SG Length**	1306.82**±**16.57 (N = 123)	1783.19**±**25.26* (N = 105)	1600.47**±**28.45* (N = 83)
**SG Width**	194.57**±**2.31 (N = 123)	268.98**±**4.16* (N = 105)	220.05**±**4.12* (N = 83)
**Polytene nucleus diameter**	46.41±0.48 (N = 100)	60.34±1.18* (N = 100)	53.79±2.68* (N = 100)

* P<0.001) when compared with corresponding control.

### Differential effects of AR or RS feeding during larval or adult stage on fecundity

**Figure 4:**
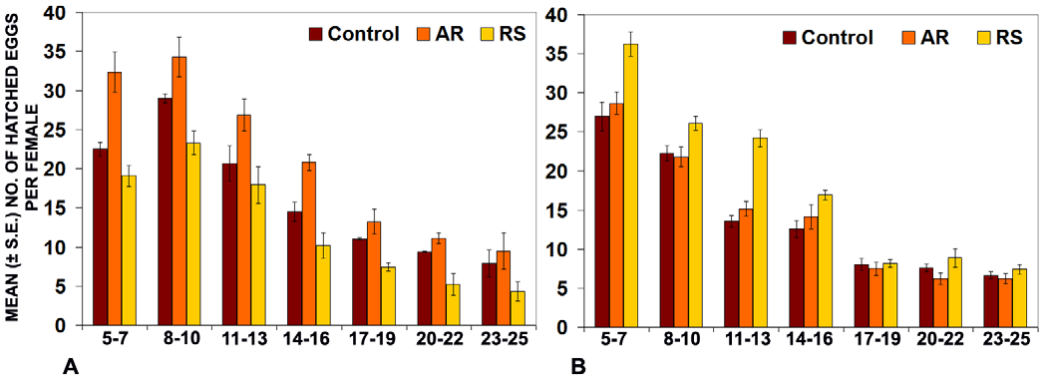
Fecundity of wild type flies (expressed as mean (±S.E.) numbers of hatched eggs on Y-axis) per female on given days (X-axis) reared on regular or 0.5% AR- or RS- supplemented food through larval and adult stages (A) or fed on 0.5% AR or RS only during the adult stage (B). For convenience of presentation, each bar represents the mean (± S.E.) number of eggs per fly that hatched during a window of three consecutive days.

Synchronized 1^st^ instar larvae were fed on regular (control) or 0.5% AR or RS supplemented food till pupation and the emerging flies were transferred, respectively, to regular or formulation supplemented food. Eggs laid by these females were monitored daily between day 5 and 25 after emergence (Fig. 4A, Table 4). In another set, larvae were reared on regular food and the freshly emerged flies were transferred to regular food (control) or 0.5% AR or RS supplemented food and eggs laid by these females were monitored as in the 1^st^ set (Fig. 4B and Table 4). Flies and larvae reared on 0.5% AR supplemented food laid significantly increased number of total and hatched eggs per female fly but those fed on 0.5% RS produced reduced number of eggs (total as well as those that hatched, Fig. 4A). On the other hand, when only the flies were fed on the AR or RS supplemented food, those receiving RS, showed significantly enhanced fecundity since the total numbers of eggs per female as well as those hatched were significantly higher than in parallel controls (Fig. 4B). Interestingly, feeding on AR only during the adult stage did not result in any noticeable change in the numbers of total or hatchable eggs laid (Table 4, Fig. 4B).

**Table 4:** Differential effects of AR or RS feeding during larval or adult stage on fecundity.

Feeding on formulation	Food	Mean (±S.E.) total no. of eggs per female in 25 days			N
		Hatched	Unhatched	Total	
**A: Larval and adult stages**	Control	115.1±0.9	40.8±0.7	155.9±1.0	2 replicates, each with 90 females and 60 males
	AR 0.5%	148.2±1.8*	39.2±0.8**	188.6±1.5*	
	RS 0.5%	87.5±1.4*	30.2±0.8*	117.7±1.1*	
**B: Only adult stage**	Control	97.7±0.9	42.2±0.7	139.8±1.3	2 replicates, each with 90 females and 60 males
	AR 0.5%	100.0±1.1**	43.7±1.1**	143.4±3.5**	
	RS 0.5%	127.9±0.9*	40.0±0.9**	167.4±1.1*	

* P<0,001 when compared with corresponding control.

** P>0.05 when compared with corresponding control.

### Feeding on AR or RS supplemented food improves thermotolerance

With a view to see if feeding on AR or RS supplemented food affects thermo-tolerance, we exposed 100 hour old *Oregon R^+^* larvae or 3-day old flies raised on regular or 0.5% AR or 0.5% RS supplemented food to different conditions of thermal stress and monitored their survival.

Late 3rd instar larvae (100 hr after hatching) reared on regular (control) or 0.5% AR or RS supplemented food were exposed to different regimens of heat shock (37°C for 60, 90 or 120 min, or at 38°C for 60 or 90 min or at 39°C for 30 min, see Table 5) following which they were restored to their respective food plates and allowed to develop at 24°C. Numbers of pupae surviving up to 72 hr after the thermal shock and those that later hatched as flies were counted. Wild type larvae reared on regular food are sensitive to a severe heat shock (e.g., exposure to 37°C for 60 min or longer or 30 min or longer at 38°C or at 39°C) and show a temperature and duration-dependent mortality (Table 5). In contrast, significantly larger numbers of those reared on either of the two formulation-supplemented food survived the milder (90 or 120 min at 37°C) as well as the more severe (38°C and 39°C) heat shock (Table 5). It is significant that while all of the larvae reared on regular food died soon after the 30 min exposure to 39°C, 6% and 17% of those reared on AR or RS supplemented food, respectively, pupated and survived at least for 72 hr after the severe heat shock. However, none of them emerged as adult flies (Table 5). Interestingly, the thermotolerance of RS-fed larvae was better than that of the AR-fed larvae under all conditions of the thermal stress to larvae (Table 5).

**Table 5:** AR or RS fed 100 hr old larvae show significantly improved survival following severe thermal stress.

Heat shock	Time (min)	% surviving 72 h after HS			% eclosion after heat shock			N
		CON	AR 0.5%	RS 0.5%	CON	AR 0.5%	RS 0.5%	
**37°C**	**60**	97.0±1.4	96.6±0.9**	97.5±1.1**	95.6±0.9	96.0±1.4**	96.5±0.8**	200
	**90**	67.2±0.9	84.7±1.5*	93.3±1.0*	25.1±1.8	47.2±1.7*	50.0±1.2*	200
	**120**	41.0±1.2	79.2*±2.1	87.8±3.1*	20. 6±2.0	43.7±3.6*	48.7±2.4*	200
**38°C**	**60**	7.5±1.3	30.5±2.3*	38.5*±1.1	4.34±0.3	6.7±0.5*	8.7±0.2*	200
	**90**	5.3±3.6	36.0±5.3*	61.3±4.8*	2.0±0.5	5.0±0.3*	6.0±0.5*	200
**39°C**	**30**	0	6.3±4.1*	17.0±5. 7*	No eclosion			200

* Values significantly different (P<0.001) from the corresponding control values.

** Values not significantly different from corresponding control values.

Three day old adult flies, reared from 1^st^ instar stage on normal (control) or AR or RS-supplemented food were exposed to 38°C or 39°C in vials which allowed monitoring of the flies during the stress. It is known that when exposed to these temperatures, the flies get knocked down or paralyzed to a motionless state from which they may or may not recover, depending upon their sensitivity and severity of the stress [20], [21]. Therefore, flies in each vial were carefully watched and the numbers of flies that were paralyzed and, therefore, knocked down to bottom of the tube during exposure to the thermal stress were counted at 15 min intervals. As the data in Table 6 show, increasing duration and temperature resulted in a proportional increase in flies that got knocked down or paralysed. It is significant that flies reared, since 1^st^ instar larval stage, on the AR or RS supplemented food showed significantly reduced incidence of paralysis after 45 or 60 min of exposure to 38°C. Further follow up of the recovered flies revealed that a significantly greater proportion of flies were alive in the AR- as well as RS-fed samples 24 hr after the 60 min exposure to 38°C (Table 6). Exposure to 39°C resulted in nearly all flies getting knocked down in all samples, especially after 30 min. It is, however, significant that in this case also, a greater proportion of flies that were reared on AR or RS supplemented food survived at least for 24 hr after the 30 min exposure at 39°C. These results clearly show that AR as well as RS feeding makes the larvae and flies more thermo-resistant.

**Table 6:** *Oregon R^+^* flies reared on AR or RS supplemented food show significantly improved thermotolerance in the knockdown assay.

		% flies knocked down			% flies surviving after 24 hr			N
Heat Shock	Time (min)	CON	AR 0.5%	RS 0.5%	CON	AR 0.5%	RS 0.5%	
**38°C**	**15**	0	0	0	68.6±1.2	87.4±1.6*	88.4±2.6*	300
	**30**	0	0	0				
	**45**	56.7±1.92	27.9±0.8*	18.5±1.3*				
	**60**	76.0±2.7	56.0±0.6*	42.6±1.7*				
**39°C**	**15**	99.0±1.0	90.2±1. 6*	87.0±1.3*	27.0±0.9	43.0±1.7*	56.1±1.4*	200
	**30**	100	100**	100**				

* Values significantly different (P<0.001) from the corresponding control values.

** Values not significantly different from corresponding control values).

### 0.5% AR- but not RS-feeding improves starvation tolerance

Flies reared on regular, AR or RS supplemented food since 1^st^ instar stage were subjected to starvation stress and the LT_50_ was estimated in each case. As the data in Table 7 show, those reared on 0.5% AR supplemented food survived much longer than those fed on regular or 0.5% RS supplemented food (Table 7). Starvation tolerance of flies reared on 0.5% RS supplemented food since 1^st^ instar stage remains comparable to those reared on regular food (Table 7).

**Table 7:** Flies reared on AR-supplemented food show greater tolerance to starvation.

LT_50_ (in hours)		
CON	0.5% AR	0.5% RS
56.8±2.2	70.5±2.0*	54.3±2.5**

* Values significantly different (P<0.001) from the corresponding control values.

** Values not significantly different (P>0.05) from the corresponding control values.

Starvation tolerance of flies reared on food supplemented with honey and Ghee or only Ghee or only honey or only the triturated Amalaki powder was similar to that of normally fed larvae (data not presented).

### Feeding on 0.5% AR or RS supplemented food during larval period enhances levels of heterogeneous nuclear ribonucleoproteins (hnRNPs)

The improved thermotolerance of formulation-fed larvae and flies may be a consequence of cellular stress caused by the dietary AR or RS since it is known that a milder stress significantly improves tolerance to a subsequent more severe stress [21], [22]. One of the very sensitive indicators of cell stress in flies is the dramatic change in the nuclear distribution of the hnRNP family of proteins, which rapidly move away following cell stress from their normal locations on active chromatin and nucleoplasmic storage sites to the chromosomal site of the *93D* or *hsrω* gene [23], [24], [25]. Members of the conserved hnRNP family of proteins have important roles in gene expression, RNA processing and transport in eukaryotic cells and thus affect cell physiology in many different ways [26]. Therefore, we examined the sub-cellular distribution and levels of three different hnRNPs, viz., Hrp36 (also known as Hrb87F), Hrp38 (Hrb98DE) and Hrp40 (Squid) in SG of larvae that were fed on normal or AR- or RS-supplemented food.

**Figure 5:**
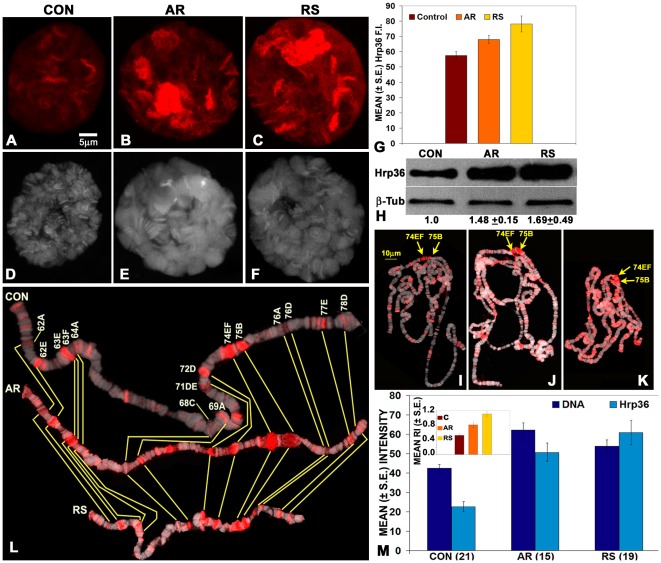
Feeding larvae on AR or RS supplemented food enhances the levels of Hrp36 in larval tissues. **A**–**F** Confocal projections show GFP (red, **A–C**) and DAPI (white, **D–F**) fluorescence in SG polytene nuclei from 110 hr old *HRB87F-GFP/Hrb87F-GFP* larvae. Histograms in **G** show that the mean levels of nuclear Hrp36 (GFP fluorescence) in polytene nuclei of SG (N = 100 for each sample) are significantly greater in AR or RS-fed larvae. Western blot in **H** shows the relative amounts of Hrp36 (detected by the P11 antibody) in total proteins from differently fed (control, AR and RS lanes) late 3^rd^ instar larvae; the relative value of Hrp36 (ratio of the signal for Hrp36 and β-tubulin in a given blot) in normally fed (1^st^ lane) larvae in each replicate was taken as 1. The normalized values of the mean (±S.E., N = 3) relative levels of Hrp36 in total larval proteins are noted below the AR and RS lanes. **I**–**K** are confocal projections of squashed SG polytene chromosomes from 110 hr old control (**I**) or AR- (**J**) or RS- (**K**) fed larvae immunostained with P11 antibody to localize Hrp36 (red fluorescence) and counterstained with DAPI (white fluorescence); the early ecdysone puffs at 75B and 74EF are marked in each case. **L** shows representative cut outs of the left arm of chromosome 3 (3L) from squash preparations as in **I**–**K** from salivary glands of 110 hr old larvae fed on regular (CON) or AR or RS supplemented food; some of the known puff sites [29] are marked on the top panel and the corresponding sites in the other two cut outs (middle and lower panels) are indicated by connecting yellow lines (the chromosome arm in these images was cut and re-positioned, as required, to provide a relatively straight orientation). Histograms in **M** show the mean fluorescence intensity for DNA and Hrp36 on the entire 3L in differently fed (noted on X-axis) larval salivary gland squash preparations as in **I**–**K**; inset shows the relative levels of Hrp36 in relation to the nuclear DNA content in the three sets of nuclei; the numbers of 3L examined for each sample is mentioned in parenthesis on the X-axis. The scale bar in **A** (5 µm) applies to **A**–**F**, while that in **I** (10 µm) applies to **I**–**K**.

*In situ* distribution of Hrp36 in intact SG cells in *Hrb87F-GFP* larvae reared under different feeding regimens was examined by confocal microscopy. The Hrp36 in SG was present in nucleoplasm and on specific chromatin regions in all the three cases (Fig. 5A–L). Interestingly, the SG nuclei from either of the formulation-fed larvae showed much higher nuclear GFP fluorescence, especially at certain chromatin regions than in corresponding tissues from larvae reared on regular food. Although the very intense signal from certain chromatin regions in nuclei from formulation-fed larvae appeared reminiscent of the strong presence of hnRNPs at the 93D site in heat shocked cells [27], a significant difference from the stressed cells is the continued presence of Hrp36 on other chromosome regions and in nucleoplasm (Fig. 5A–F). Parallel observations on polytene chromosome spreads revealed that the chromosome sites showing very intense Hrp36-GFP fluorescence in intact glands actually correspond to the early ecdysone puffs at 74DE and 75B on 3L, rather than the 93D puff (see below and Fig. 5L). Measurement of total nuclear GFP fluorescence in the distal salivary gland nuclei clearly showed that the levels of nuclear Hrp36 are significantly increased in AR- or RS- fed larvae (Fig. 5G). Comparison of the levels of Hrp36 in total proteins from *Oregon R^+^* larvae by western-blotting using the Hrp36-specific P11 antibody [28], confirmed that the relative levels of this proteins were significantly higher in the body of formulation-fed larvae (Fig. 5H).

In order to identify the specific chromosome regions which show very high presence of Hrp36 in SG nuclei from formulation fed larvae, we immunostained polytene chromosome spreads from 110 hr old larvae, that were reared on normal, AR- or RS-supplemented food, with the Hrp36-specific P11 antibody [28]. Our observations showed that unlike the very high level of the hnRNPs seen at the stress induced 93D puff in conventionally stressed cells with concomitant absence from almost all other chromosomal sites [27], [28], the formulation feeding did not qualitatively alter the chromosomal locations of hnRNPs. However, the amount of these proteins on different puff and other sites was significantly higher in SG from larvae fed on 0.5% AR or RS- supplemented food than in those from larvae reared on regular food (Fig. 5I–K). A comparison of the site-wise distribution of Hrp36 on the left arm of chromosome 3 (3L) in polytene spreads from differently fed larvae (Fig. 5L) clearly revealed qualitative similarity in distribution of the Hrp36 on various developmental puffs. Significantly, however, the levels of Hrp36 present at each of these puff sites were much higher on chromosomes from formulation-fed larvae than in larvae reared in parallel on regular food (control). The stage of larvae used for these studies corresponds to puff stage 10 described by [29], which coincides with the activation of early ecdysone-responsive puffs like the 74EF and 75B in SG (Fig. 5H–J, L). Accordingly, and in agreement with an earlier report [30], a high level of Hrp36 was present at these puff sites. It is very interesting that there is a substantially greater presence of Hrp36 at the 74EF and 75B puffs in the formulation-fed larvae (Fig. 5I–K, L). Measurement of DAPI fluorescence (DNA content) and the P11 immuno-fluorescence (Hrp36) of the entire 3L showed (Fig. 5M), in agreement with the data on total nuclear DNA content presented in Table 2, that the amounts of DNA as well as Hrp36 were significantly greater in 3L from the formulation-fed larvae. Interestingly, a comparison of the amount of Hrp36 on per unit DNA on 3L (relative intensity shown in inset in Fig. 5M) revealed that compared to the increase in DNA content, the increase in Hrp36 on different chromosome regions is significantly greater, which shows that the increase in Hrp36 cannot be explained only by the increase in DNA content in formulation-fed larval polytene chromosomes. There is a net increase in the chromosome associated Hrp36 in formulation-fed larvae. Analysis of the distribution of Hrp36 on the right arm of chromosome 3 (3R) in these squash preparations showed results similar to those for the 3L (details not shown).

**Figure 6:**
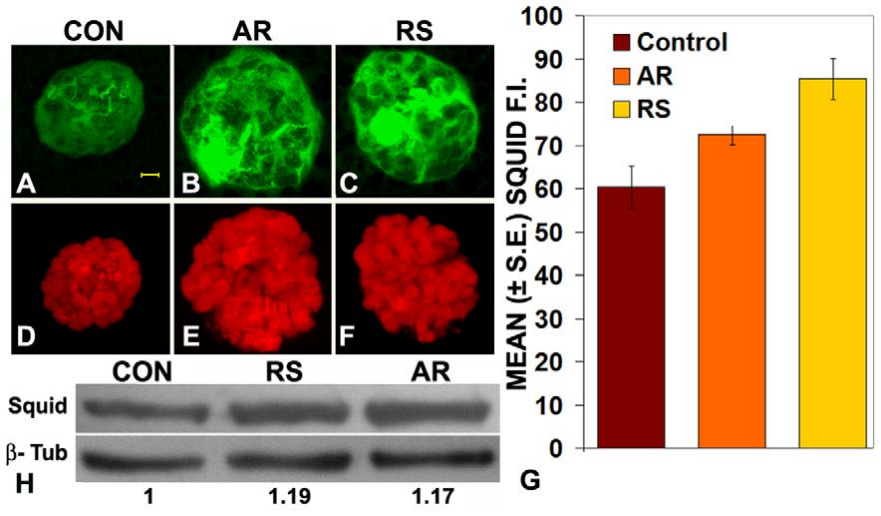
Levels of Hrp40 (Squid) are increased in formulation fed *Squid-GFP* larvae. Confocal projection images of GFP fluorescence (green, **A**–**C** and **G**–**I**) in polytene nuclei from late third instar larval salivary glands (**A**–**C**); DAPI-stained chromatin (**D**–**F**) is seen in red. Histograms in **G** show mean intensity of Squid-GFP fluorescence in SG polytene nuclei (N = 100 for each sample) from differently fed larvae. Western blot in **H** shows relative levels of Squid-GFP protein (Squid) and β-tubulin (β-tub) in whole proteins from differently fed (CON, RS or AR) larvae challenged with GFP antibody: the values below each lane indicate the relative levels (ratio of Squid∶β-tubulin in the blot with the value for larvae reared on regular food (CON) taken as 1.0) of Squid-GFP protein. The scale bar in **A** represents 5 µm and applies to **A**–**F**.

To determine the cellular levels of the Hrp40 (Squid) protein, we used the *Squid-GFP* protein-trap allele so that the sub-cellular distribution of the Squid protein can be monitored through the GFP fluorescence. Confocal microscopic examination of GFP fluorescence in SG from late 3^rd^ instar *Squid-GFP* larvae reared since hatching on regular (Control) or 0.5% AR or RS supplemented food revealed significantly increased levels of Squid in the formulation-fed larvae, with several puff regions showing massive accumulation of Squid-GFP (Fig. 6A–F) in a manner comparable to that noted above for the Hrb87F protein (Fig. 5). Comparison of mean GFP-fluorescence intensity of distal polytene nuclei in SG from differently fed larvae (Fig. 6G) confirmed that the nuclear levels of Squid protein are indeed enhanced in larval tissues from the formulation fed larvae. Detection of Squid-GFP in total larval proteins by western blotting (Fig. 6H) also confirmed this protein's elevated levels in formulation-fed larvae.

The Hrb98DE (Hrp38) also shows a comparable increase in its levels in formulation-fed larval tissues (details not presented). Examination of Hrp36, Hrp38 and Hrp40 proteins in Malpighian tubules too showed a comparable increase in levels of these proteins in larvae that were fed on 0.5% AR or RS-supplemented food (not shown).

A summary comparison of the different effects of AR or RS supplemented food on the parameters examined in this study is presented in Table 8.

**Table 8:** Comparison of the observed effects of dietary supplement of AR or RS on different parameters in *Drosophila melanogaster*.

Parameter	Rearing on 0.5% AR supplemented food	Rearing on 0.5% RS supplemented food
Median life span	Increased	No effect
Developmental time	Shorter	Shorter
Larval salivary gland size and polytene level	Larger glands with additional cycle/s of endo-replication	Larger glands with additional cycle/s of endo-replication
Fecundity	Improved with larval feeding but no effect of adult feeding	Decreased with larval feeding but improved with adult feeding
Thermo-tolerance	Improved	Better than with AR-supplement
Starvation tolerance	Improved	No effect
Nuclear hnRNP levels in larvae	Increased, including on puffs	Greater increase than with AR-supplement

## Discussion

We tested two different categories of formulations, one being a *Rasayana* (*Amalaki Rasayana*) and the other a *Bhasma* (*Rasa-Sindoor*), for their effects on longevity, development, fecundity and stress-tolerance of *Drosophila melanogaster*. Both the formulations were prepared essentially as classically described with some modifications to ensure reproducibility and proper hygienic conditions. It may be noted that the available tools and preparatory methods have inevitably changed with the passage of centuries since the classical texts were written. This has also involved local variations in preparatory procedures and nomenclature of the formulations. The traditionally described elaborate practice of “*sandhanam*” which requires the AR mixture to be preserved in Ghee smeared vessel under ashes for one year and addition of other ingredients like sugar, *Pippali* etc were avoided in our method of preparation of AR to ensure reproducibility and hygienic conditions as required in current practices. Nevertheless, these two formulations were prepared essentially following the traditional practices. HPLC analysis (data not presented) confirmed that the different batches of the formulations were comparable in their constitution. It may also be noted that these batches of AR and RS were prepared solely for the coordinated research work directed to investigate and understand the scientific basis of Ayurveda [4]. The same AR has been used in the study of Swain et al [40] on effect of AR feeding on genomic integrity in rat brain.

A comparison of the observed effects (see Table 8) reveals that the two formulations affected life-history and other physiological traits in distinct ways. While the size of larval salivary glands, levels of hnRNPs in larval tissues and thermotolerance of larvae/adult flies improved following rearing on food supplemented with either of the two formulations, the median life span and starvation resistance improved only with AR-feeding. Interestingly, feeding on AR or RS improved fecundity, but the developmental stage at which the formulation was effective varied. Thus while larval and adult feeding with AR increased the fecundity, feeding larvae and adults with RS had the opposite effect. On the other hand, feeding only during the adult stage with AR supplement had no effect on fecundity but the RS-feeding during the same stage improved it. Such specific but different effects of the two Ayurvedic drugs tested in this study show that the observed effects are consequences of specific changes in the organism's metabolic activities following the dietary AR or RS supplement. Interestingly, the observed effects seem to generally agree with the reported usages of the two formulations in human [13].

Fruits of Amla or Indian gooseberry, the principal component of AR, are known to be very rich in anti-oxidants as revealed in several studies on different extracts of these fruits [31], [32], [33], [34], [35], [36]. However, effects of complete AR on life history traits have rarely been examined. Two recent reports have claimed that dietary provision of a “modified” AR dramatically enhances longevity of flies [37], [38]. Compared to the near doubling of life span claimed in these two publications, the increase in median life span observed in our study following 0.5% AR feeding is modest. It is, however, to be noted that these two reports [37], [38] are based on rather small sizes of samples of control and formulation-fed flies; moreover, these studies do not provide the required details of the actual formulation used and the quantity provided to flies. Therefore, results claimed in these studies remain uncertain [39]. It is possible that some variations in the dietary delivery of AR may show more pronounced effects on longevity than observed in the present study. The significant point, however, is that feeding on 0.5% AR supplemented food enhanced the median life span of experimental flies. As noted earlier, one of the major usages of AR in human is to improve longevity and youthfulness. A recent study [40] has shown that the genomic integrity in ageing mice provided with AR is significantly better than control sibs.

It is interesting that higher concentrations of RS marginally reduced longevity of flies while 0.5% or lower concentrations of RS did not affect the median life span. In this context it is significant to note that although RS has varied applications in Ayurvedic treatment procedures [12], it is not claimed to improve longevity in human. RS is essentially mercuric sulphide with some organic components. Mercury salts are generally reported to be highly toxic and carcinogenic [13], [41], [42], [43]. However, very few studies have examined the *in vivo* biological effects of *Rasa-Sindoor*. The mineral materia medica of Ayurveda claims mercury to have the power to assimilate (*rasanate*) all other metals [2]. A significant finding of our study is that feeding on RS-supplemented food did not elicit any evidence of heavy metal toxicity in larvae or flies since there was neither any evidence of lethality following RS-feeding nor of any developmental defects in the emerging flies. Supplementing food with 1% or higher concentration of RS resulted in a slightly reduced life span, but no developmental abnormalities or phenotypic consequences were noticed. Thus it appears that the processing of mercury in the specified Ayurvedic manner, which involves a systematic sublimation and grinding (see Materials and Methods), seems to convert the mercury sulphide to nano particles [14]. It may be noted that cinnabar, which too contains mercury sulphide, is widely used in traditional Chinese medicines [44]. Another recent study has also shown that the human gut flora does not convert mercury sulphide present in cinnabar into toxic derivates like methylmercury [45].

The observed differences in effects of the two formulations on fecundity are interesting. It is known that good larval nutrition/growth is associated with improved fecundity [46], [47]. The anabolic effects of AR are reflected in faster larval development and increased size and polyteny levels in larval salivary glands. The overall elevated levels of hnRNPs, including those bound to active chromosome regions, also appear to reflect more robust gene expression patterns in the AR-fed larvae. All these may prepare the larval ovaries to produce more eggs during the adult stage. On the other hand, providing AR only during the adult stage fails to alter the state of gonads as established during the larval period. In this context, it is intriguing that RS had adverse effect on fecundity when provided during larval period but significantly improved fecundity when provided only during the adult stage. The physiological bases for such contrasting effects on fecundity need further cellular and molecular studies but it is interesting to note that in Ayurvedic practice, RS is not recommended for growing children but is indicated, among other things, for genital disorders and rejuvenation in adult subjects [13].

The significantly greater thermotolerance shown by larvae and adult flies that were reared on AR or RS-supplemented food is remarkable. It is known that a prior exposure to a mild stress makes the cells/organism better thermo-protected [21], [22], [48], [49]. Therefore, we examined the possibility that these two formulations, especially the mercury based RS, may cause a mild/chronic stress which may result in improved tolerance to a more severe subsequent stress. Several studies in *Drosophila* have shown that heat shock brings about a rapid and dramatic change in the distribution of nucleoplasmic and chromosome associated hnRNPs so that soon after heat shock, almost all of them get aggregated at the 93D or *hsrω* locus [23], [27], [28], [50]. Our present observation on the *in situ* distribution of three different hnRNPs, viz., Hrp36, Hrp38 and Hrp40, do not provide any evidence for the AR- or RS-fed larval cells being under stress since in all cases, the nuclear distribution of the three hnRNPs was qualitatively similar to that seen in normally fed larval cells. Heat shock does not increase the quantity of hnRNPs in cells [25] but the formulation-fed larval cells displayed significantly enhanced levels of these proteins, which as mentioned above, seems to reflect more robust developmental gene expression. Therefore, the improved thermotolerance of the formulation-fed larvae/flies is apparently not due to a mild stress inflicted by the formulations. The abundance of anti-oxidants in Indian gooseberry fruit [31], [32], [33], [34], [35], [36] may partly explain the high thermotolerance of larvae/flies reared on AR-supplemented food but so far there is no evidence that RS also has similar properties. Studies are in progress to see if the induction of different heat shock proteins is modulated by the dietary AR or RS.

The improved starvation-tolerance of AR-fed larvae/flies agrees with earlier reported [46], [47] correlation between better larval physiology and longevity of adults with starvation tolerance. In this context it is significant that while RS-feeding also improved the larval physiology, in terms of developmental time, salivary gland size and enhanced levels of hnRNPs, it had no effect on fly longevity and starvation tolerance.

The hnRNPs are a highly conserved group of primarily nuclear proteins that dynamically bind with the RNA-pol II transcribed nascent transcripts but do not stably associate with other RNA-protein complexes [51]. Mammalian cells have about 30 different hnRNPs, which are grouped into distinct sub-families; *Drosophila*, on the other hand, seems to have less diverse repertoire of these proteins [26]. The hnRNPs are involved, in conjunction with some other classes of RNA-binding proteins, in the synthesis and subsequent processing (splicing, capping and polyadenylation) of the pre-mRNAs, as well as in transport of the final mRNA to their destination [26]. Several of these functions are carried out by more than one hnRNP members in a combinatorial mode which results in a “RNA splicing code” [52]. In the present study we found that nuclear levels of the three examined members of hnRNP A/B family (Hrp36, Hrp38 and Hrp40) were significantly enhanced in AR or RS-fed larvae. It has been reported earlier that over- or under-expression of one hnRNP can adversely affect certain cell activities, although redundancy in functions of some of the hnRNPs acts as a compensating buffer system [26], [52], [53], [54], [55]. It is significant that either of the two Ayurvedic formulations enhanced the levels of all the three hnRNPs comparably so that activities that are dependent upon combinations of specific hnRNPs [9], [52] are not adversely affected because of alterations in their relative abundance. We believe that elevation in levels of the hnRNPs in formulation-fed larvae provides for more robust developmental gene expression and RNA-processing etc. so that the overall physiology of the organism is better, as reflected in enhanced polyteny levels, slightly faster development and improved stress-tolerance. In this context, it is interesting to note that Hrp36-null mutants display similar larval or pupal development time irrespective of their rearing on normal or AR or RS-supplemented food (V. Dwivedi and S. C. Lakhotia, unpublished). This suggests that the enhanced levels of hnRNPs in the formulation-fed larvae are indeed involved in their faster growth.

Many earlier studies using *Drosophila melanogaster* have identified quantitative traits like longevity, development time, body/organ size, fecundity and starvation tolerance as indicators of individual and population fitness and these parameters have been utilized to study evolution in laboratory populations under selection for one or the other of these parameters to determine the tradeoffs between these measures of fitness [47], [56]. Thermotolerance is also affected in a complex manner by quantitative trait loci (QTL)-linked genes [57]. It is obvious that the relationship between the different life history parameters is complex and therefore, specific experimental conditions may produce divergent results [34]. Although in our studies we did not examine any long-term population effects, the qualitative and quantitative differences in responses to the two Ayurvedic formulations within the same generation agree with the existence of complex regulatory networks that influence the diverse life history parameters. Notwithstanding the complexity of trade-offs between the different life history traits like longevity, fecundity and stress-tolerance, it is clear that these life history traits, which have bearing on individual's fitness, are affected in a manner largely comparable to that claimed in human.

A significant finding of the present study is that unlike the complete AR formulation, its individual ingredients, viz., Ghee or honey, when supplemented separately did not affect development time or starvation- or thermo-tolerance. Supplementing only the triturated Amalaki powder had only a marginal effect on development time but none on starvation- or thermo-tolerance. It is to be noted that the honey and Ghee are added to the triturated Amalaki powder as *Anupana*. Our results agree with the Ayurvedic principle that *Anupana* as a medium of administration improves acceptability and palatability and helps in action of the main drug.

In summary, our present results establish that, as for many other studies of relevance to human health and drug discovery [5], [6], [7], [8], [9], [10], *Drosophila melanogaster* is a very good model for understanding the scientific bases of actions of different traditional formulations, like those of Ayurveda. Subsequent studies would be directed to understand the molecular and cellular bases of the observed effects on life-history and other traits. The very rich repertoire of mutants and other genetic analysis systems available in fly genetics would, in future studies, enable in-depth cellular and molecular analyses of the effect/s of the given Ayurvedic formulation. The presently available and emerging strategies in the fly model would enable specific studies on effect/s of the given formulation on general physiology, anti-oxidant and DNA repair status, stress-tolerance, protein-quality control, immune response, developmental plasticity etc. We expect that exploitation of the fly system with unbiased scientific rigour would help us understand the mechanistic details of actions of the different traditional medicinal systems and thereby improve their management and applications.

## Materials and Methods

### Preparation of formulations

The two formulations, viz., *Amalaki Rasayana* and *Rasa-Sindoor* were prepared at the Arya Vaidya Sala, Kottakkal, following the traditional methods as described below.

#### Amalaki Rasayana

Preparation of AR requires four steps. Dried gooseberry fruit was pulverised using Tyco Pulveriser (step 1) and parallely, gooseberry juice was prepared from fresh fruits with a juice extractor (step 2). In the 3^rd^ step (trituration), products of the steps 1 and 2 were blended in 1∶1 ratio and dried for about 24 hours at 55°C under low pressure (700 mm) in a Vacuum Tray Drier. The dry mass thus formed was pulverised and the steps 2 and 3 were repeated another 20 times (trituration for 21 times). Quantity of the additional gooseberry juice added at each trituration step remained the same as in the 1^st^ trituration. These processing steps take about 2 to 3 months to complete. The AR was finally prepared at step 4 by blending the dry Amalaki powder (obtained after completion of 21 cycles of trituration) with commercially available honey (M/s. Hexa Apiarium Pvt. Ltd., Kannur, Kerala, or M/s. Galaxy Honey, Kottakkal, Kerala) and Ghee (“Milma” from Malabar Milk Marketing Federation, Govt. of Kerala, Kozhikode, Kerala or “Nandini” from Karnataka Milk Marketing Federation, Govt. of Karnataka, Mysore, Karnataka) in a 1∶2∶0.5 ratio resulting in a thick sticky paste.

Amla fruit and powder were authenticated by Centre for Medicinal Plants Research, Kottakkal and the Quality Assurance (QA) department of Arya Vaidya Sala (AVS), both of which are approved by Govt. of India for testing and issuing quality control certificates for Ayurvedic raw materials and finished products. Macroscopic/organoleptic (shape and taste of fruits) and microscopic parameters (nature of pericarp, mesocarp and vascular bundles of fruits) were compared with the standard values specified in Ayurvedic Pharmacopoeia of India (API). In addition, in-house developed HPTLC profiles of (a) raw Amalaki fruits, (b) Amalaki powder, (c) in-process samples at the conclusion of 1^st^, 10^th^, 15^th^ and 21^st^ steps of trituration and (d) the finished formulation were also compared with the standard HPTLC profiles of gallic acid and ellagic acid. Quality control assays on raw-materials and HPTLC profiles provided evidence for consistency of different batches used in this study. The final formulation was packed in sealed 45 g HDPE containers and stored under ambient conditions. HPTLC analysis indicated the shelf-life of finished product to be 12 months and, accordingly, each batch was used within this period.

#### Rasa-Sindoor

Preparation of RS also involves four steps. In step 1, sulphur was pre-processed in a three stage procedure in which sulphur and Ghee (1∶1) were mixed and melted over fire. The molten mixture was mixed with milk (twice the quantity of sulphur). When cooled, the mix was drained and the residue repeatedly rinsed in water. The washed sulphur was subjected to the above three stages twice again. The pre-processed sulphur was dried by mild heating. Step 2 involved the mixing of pre-processed sulphur, mercury and *Aloe-vera* juice [12] in 1∶1∶0.7 ratio and grinding of the mixture in an electric wet grinder (85 RPM) for about 240 hours until the mixture became a thin paste, which is called *kajjali*. In step 3, the *kajjali* was spread on a wooden plank and kept for drying under shade. After about four days' time, the dry mass was pulverised to a fine powder in domestic Mixer. In step 4, this powder was taken in a porcelain bowl whose mouth was covered with another inverted porcelain bowl of the same size. The junction of the two bowls was sealed with cloth smeared with wet clay. The twin bowls were thus made into a sealed sphere by complete coverage with clay smeared cloth. After drying of the clay, the sphere was charged into an open-hearth furnace and heated up to 620°C for 48 hours. Thereafter the bowl sphere was allowed to cool and the clay and clothing cover were removed and the two bowls were slowly separated. The *Rasa-Sindoor*, which is the sublimed product, sticks to the inner surface of the upper bowl. This sublimed deposit was carefully scraped out as flakes and further pulverised by grinding in a mortar and pestle. The final product is a fine dust of shining brick red colour. Appropriate chemical assays were carried out to check quality control of the RS preparations.

The quality control certificate for RS by the QA department of AVS was based on the following Pharmacopoeial parameters: (a) test for Hg and S, (b) assay of Hg and S, (c) acid insoluble ash, (d) water soluble ash and (e) loss on ignition at 500°C for 30 min. Additionally, the typical Ayurvedic parameters like *Nishchandrika* (lustreless), *Rekhapurnatwam* (fineness), *Varitaritwam* (water floating), etc were also applied. In conformity with the stoichiometry of HgS, mercury in the final preparation was 84.8% w/w while sulphur was 15.6% w/w. Ayurvedic Pharmacopoeia of India specifies the shelf life of RS as infinite since HgS (Cinnabar) is known to be highly stable. For the present set of studies, the final RS powder was packed and stored in 100 g sealed containers and used within a period of 12 months. All the test and assay reports for AR and RS are available with AVS, Kottakkal.

### Fly rearing and food preparation

*Oregon R^+^* (wild type) and other fly stocks were maintained under uncrowded condition at 24°C±1°C. All studies on the effects of the dietary supplements on life-history traits were carried out with the *Oregon R^+^* flies while for some of the experiments to assess the status of heterogeneous RNA-binding proteins (hnRNP), the Hrb87F-GFP, Hrb98DE-GFP and Squid-GFP protein-trap lines [58], [59] were used. In these protein-trap transgenic lines, the endogenous Hrb87F (Hrp36) or Hrb98DE (Hrp38) or the Squid (Hrp40) protein, respectively, gets tagged with GFP so that the sub-cellular distribution level of the given protein can be monitored through the GFP fluorescence. All the three GFP-tagged proteins function more or less like their original native proteins.

Flies were fed on standard fly food containing agar, maize powder, yeast and sugar. The AR or RS formulation was added at the desired concentration (weight/volume, see Results) to freshly prepared fly food while it was still in fluid state and thoroughly mixed with a glass rod. The mixed food was poured into containers (bottles, vials or Petri plates, as required) and allowed to cool and solidify before use. In some experiments, the fly food was supplemented with desired concentration of either a mixture of honey and Ghee (in 2∶0.05 ratio) or honey or Ghee or the Amalaki powder (as obtained after 21 cycles of trituration) alone.

In all formulation feeding experiments, eggs were collected from fly stocks that had always been reared on regular food. For each experiment, the regular and the formulation supplemented foods were prepared from the same batch; likewise all larvae/adults for a given experiment were derived from a common pool of eggs of the desired genotype and reared in parallel on the regular or the supplemented food. The characteristic dark brownish red colour of RS was distinctly visible in the guts of larvae and adult flies. The AR-fed larvae, on the other hand, showed brownish-black colour in their gut. Thus the formulation mixed food was indeed taken up by the larvae/flies.

### Viability assay

Viability assay was carried out with freshly eclosed wild type flies reared, since the 1^st^ instar larval stage, on regular (control) or differently supplemented food at 23±1°C. Eight replicates were carried out with 25 flies per replicate. Flies were transferred, without anesthetization, to fresh vials after every 2–3 days and the number of surviving flies was recorded on daily basis until 60 days after eclosion. The median life span for each experimental condition was calculated as the day till which 50% of the original number of flies (N = 200 from 8 replicates for each feeding regime) survived in the vial.

### Developmental assay

For developmental assay, eggs were collected at 1 hour interval and the freshly hatched larvae were gently transferred to food vials containing either 0.5% AR or 0.5% RS supplemented food or to vials with the regular food (control) and reared at 23±1°C. After 4 days of development, the time at which individual larvae pupated and subsequently emerged as flies was monitored at 2 hr intervals.

### Sizes of salivary glands and polytene nuclei in formulation-fed late third instar larvae

In order to determine the sizes of larval organs or their cells in wild type larvae reared at 24±1°C on regular or AR or RS supplemented food, salivary glands (SG) were selected because these endoreplicated tissues have a defined morphology, fixed number of cells and follow a highly regulated pattern of endo-replication cycles [16], [17], [18]. In order to obtain SG of same age, larvae that had just everted their anterior pair of spiracles were selected and immediately dissected in Poels' salt solution (PSS) [60] fixed in 3.5% paraformaldehyde in phosphate buffered saline (PBS, 13 mM NaCl, 0.7 mM Na_2_HPO_4_, 0.3 mM NaH_2_PO_4_, pH 7.0), washed in PBS and stained with DAPI (4′,6-diamidino-2-phenylindole dihydrochloride, 0.5 µg/ml). The length and width of the each gland was measured using the Overlay and Histo options in the Zeiss LSM Meta 510 software. The diameter and DAPI fluorescence of 5–7 distal most polytene nuclei in each gland were also measured to estimate mean size and DNA content of polytene nuclei.

### Fecundity

For examining the effect of formulation-feeding on fecundity of flies, two sets of feeding regimen were followed. In one set, first instar larvae were transferred to regular or formulation-supplemented food to develop at 23±1°C. Freshly eclosed males and virgin females from these cultures were separated and transferred to fresh regular or respective 0.5% formulation supplemented food for three days, followed by mixing on the fourth day; the mixed flies were transferred to population cages with regular or formulation supplemented food dishes, respectively, till the end of experiment. In the 2^nd^ set, effect of feeding on formulation supplemented food only during the adult stage was examined. Freshly eclosed male and virgin female flies from larvae reared on regular food were separated and kept for three days in food vials containing the formulation supplemented food before mixing them for on the 4^th^ day and transfer to population cages carrying the respective food plates. Food plates were replaced each day with fresh regular or formulation supplemented food plates, respectively. Total numbers of eggs in each of the plates and those that hatched or did not hatch were counted daily. Number of hatched eggs per female fly was calculated by dividing the total number of hatched eggs with the total number of female flies alive during the given day.

### Thermotolerance assay

Freshly hatched *Oregon R^+^* larvae were fed either with regular or 0.5% AR or 0.5% RS supplemented food and reared at 24±1°C. 100 hr old third instar larvae were kept in microfuge tubes lined with moist filter paper and subjected to heat shock (HS) at different temperatures (37°C, 38°C or 39°C) for 30, 60, 90 or 120 min. Following HS, the larvae were gently transferred to vials containing regular *Drosophila* food and allowed to develop further. The numbers of individuals dying at subsequent stages of development were counted till the emergence of flies. In another set, three day old *Oregon R^+^* flies fed through larval stages either on 0.5% formulation supplemented or regular food were lightly etherised and kept in plastic vials (25 flies per vial). The flies were allowed to recover from anaesthesia for at least 4 hr prior to the HS at 38°C for 60 min. or at 39°C for 30 min. The number of flies which fell down during HS was monitored every 15 min. Subsequently, the flies were transferred to food vials and the numbers of flies surviving after 24 hr were recorded. Eight replicates were carried out for each feeding condition.

### Starvation resistance assay

Freshly hatched *Oregon R^+^* larvae were reared on 0.5% AR or 0.5% RS supplemented or regular (control) food at 24±1°C. Three day old flies (formulation fed and control) were starved in empty bottles containing filter paper strips soaked with water [61]. Mortality of starved flies was recorded every 12 hours. A total of 500 flies (5 replicates of 100 flies each) were tested for each group.

### Distribution and quantification of different hnRNPs (Hrp36, Hrp38 and Hrp40) in larval tissues

Levels of the Hrp36, a HnRNP-A1 homolog in *Drosophila* [62] in intact SG or in squashed SG polytene chromosomes from 110 hr old larvae reared at 24±1°C on different feeding regimes were assessed either by measuring the GFP fluorescence in individual nuclei in the *HRB87F-GFP/HRB87F-GFP* [59] larval SG or by immunostaining of polytene chromosome squashes from wild type larvae with the Hrp36-specific P11 antibody 1∶20 dilution, [28] as described earlier [25], [27]. Western-blotting was carried out with total body proteins from differently fed larvae and separated by polyacrylamide gel electrophoresis to compare the levels of Hrp36 using the P11 antibody (1∶200 dilution) as described earlier [27]. The levels of two other hnRNPs, viz., Hrp40 (hnRNP A1 or Squid) and Hrp38 (Hrb98DE) were examined using the respective protein-trap alleles (*Squid-GFP* or *Hrb98DE-GFP*) by measuring the GFP fluorescence in SG nuclei of differently fed late third instar larvae. Western-blotting was carried out with total proteins from differently fed *Squid-GFP/Squid-GFP* larvae using GFP antibody (1∶500, Sigma-Aldrich, India). In each western blot, the levels of β-tubulin, detected with the E7 β-tubulin antibody (1∶200, Developmental Studies Hybridoma Bank, Iowa), were used as the loading control. The secondary antibody for immunostaining was the Cy3 conjugated anti-mouse IgG, (1∶200, Sigma-Aldrich, India) while for western blots, HRP conjugated anti-rabbit IgG (1∶1500; Bangalore Genei, India) or HRP conjugated anti-mouse IgG (1∶1500; Bangalore Genei, India) was used.

### Microscopy and documentation

All GFP or immuno-fluorescence stained preparations were examined with a Zeiss LSM 510 Meta confocal microscope using appropriate lasers, dichroics and filters. The light/DIC microscopic examinations were carried out with a Nikon E800 microscope with appropriate filter combinations and the images were recorded with a Nikon DXM 1200 digital camera. The different objectives used for confocal or other microscopy were 10× (0.3NA, Plan Fluor), 20× (0.5NA, Plan, Fluor) or 60× oil (1.4NA, Plan Apo). All the images were assembled using Adobe Photoshop 7.0.

### Statistical analysis

Sigma Plot 11.0 software was used for statistical analyses. All replicates for a given control or experimental sets were tested for homogeneity using the Holm-Sidak method. None of them showed any significant intra-group differences. For comparison between the control and formulation-fed samples, One-Way ANOVA was performed.
